# Research progress on neuromolecular imaging of REM sleep behavior disorder

**DOI:** 10.3389/fneur.2022.1009907

**Published:** 2022-10-10

**Authors:** Chaofan Geng, Hongju Zhang

**Affiliations:** ^1^Henan University People's Hospital, Henan Provincial People's Hospital, Zhengzhou, China; ^2^Department of Neurology, Zhengzhou University People's Hospital, Henan Provincial People's Hospital, Zhengzhou, China

**Keywords:** idiopathic rapid eye movement sleep behavior disorder (iRBD), neuromolecular imaging, alpha-synuclein, conversion, sleep

## Abstract

Idiopathic rapid eye movement sleep behavior disorder (iRBD) is an important non-motor complication of Parkinson's disease. At the same time, iRBD is considered to be the prodromal stage of α-synucleinopathy. This high risk of conversion suggests that iRBD becomes a nerve It is a window for early research on degenerative diseases and is the best candidate for neuroprotection trials. A wide range of neuroimaging techniques has improved our understanding of iRBD as a prodromal stage of the disease. In addition, neuroimaging of abnormal iRBD is expected to be a potential biomarker for predicting clinical phenotypic transformation. This article reviews the research progress of neuromolecular imaging in patients with iRBD from the perspective of iRBD transforming synucleinopathies.

## Introduction

Idiopathic rapid eye movement (REM) sleep behavior disorder (iRBD) is a parasomnia mainly characterized by the loss of muscular atonia and dream enacting behaviors during REM sleep (1, 2). The pathogenesis is related to the dysfunction of the locus coeruleus and the ventral nucleus of the medulla oblongata, which have a role in regulating muscle tone during the REM period (3). Longitudinal studies have reported that 80% of clinically diagnosed iRBD patients can develop neurodegenerative disorders after 10-year follow-up, especially α-synucleinopathies, such as Parkinson's disease (PD), dementia with Lewy bodies, and multiple system atrophy (MSA) (1, 4, 5). Accordingly, iRBD is considered to be a powerful prodromal state of α-synucleinopathies (6) and has become the most important clinical symptom for predicting neurodegenerative diseases (7). However, the time course of iRBD conversion to α-synucleinopathy is highly variable (8), and the search for early biomarkers of iRBD conversion becomes a preferred problem (9), which could contribute to delaying disease progression in its earliest stages.

Currently, more and more studies are looking for early neuroimaging evidence of iRBD transformation in order to detect structural and functional abnormalities in the iRBD brain earlier (10). Among neuroimaging markers, radionuclide imaging is a widely used molecular imaging technique because of its precise localization and specific labeling (11), mainly including Positron emission tomography (PET), Single photon emission computed tomography (SPECT), and ^123^Iodine-metaiodobenzylguanidine (^123^I-MIBG) cardiac imaging.

Considering all these factors, we review the latest research on molecular imaging between iRBD and α-synucleinopathies. The molecular neuroimaging markers that predict the early transformation of iRBD were the place we pay the closest attention to in this article. A comprehensive understanding and review of neuroimaging techniques to explore phenotypic conversion in iRBD will be able to guide future research in this field and further facilitate the clinical management of this disease.

## The pathophysiology of iRBD

The exact pathophysiology of iRBD is not fully established. Animal models of RBD with damage to the sublaterodorsal tegmental nucleus (SLD) showed behavioral abnormalities during REM sleep similar to those of human RBD (12). Previous studies have confirmed that the SLD is the key brain structure that triggers muscle retardation in REM sleep and that selective blockade of glutamatergic transmission in the SLD leads to REM sleep without atonia (RSWA) (13). During REM sleep, SLD inhibits skeletal muscle movement and dystonia during REM sleep by activating direct and indirect inhibitory pathways (13). SLD neurons act on interneurons *via* a direct pathway. Intermediate neurons in turn inhibit glycinergic and gamma-aminobutyric acidergic neurons *via* spinal anterior horn motor neurons, resulting in skeletal during REM sleep skeletal muscle movements being inhibited during REM sleep (14). In addition, glutamatergic neurons in the SLD can also activate the ventral medial reticular formation (VMD) of the medulla oblongata through an indirect pathway, leading to a relaxation of skeletal muscle tone (15). Under normal waking conditions, direct stimulation of the VMM can effectively trigger REM sleep and lead to motor inhibition during REM sleep. SLD and VMM together form a complete brainstem circuit, and damage to any part of this circuit may lead to RBD (16).

## Altered intracranial metabolism in iRBD

### Changes in glucose metabolism in the brain

[^18^F] fluorodeoxyglucose (FDG) positron emission tomography (PET) is an assay targeting glucose metabolism visualization in the brain, with higher metabolic rates presenting high signal changes (17), reflecting the metabolic activity of neurons in the brain. The first ^18^F-FDG-PET scan was performed on nine patients with iRBD, and four patients were found to have decreased glucose metabolism in the occipital cortex, especially in the primary visual cortex (PVC), which is the priority area for patients with MSA. In contrast, the other five patients showed hypermetabolic changes in the left cingulate gyrus, right frontal lobe and right temporal lobe, which are the preferentially affected regions of PD patients (18). The differences in metabolic profiles between regions suggest heterogeneity of clinical conversion in patients with iRBD. Another study reported that 63. 6% of people with decreased occipital cortex glucose metabolism converted to iRBD after an average of 3 years (19), suggesting that decreased occipital glucose metabolism is an early intracerebral change in iRBD. iRBD patients also have increased glucose metabolism in the hippocampus, cingulate gyrus, pons and posterior cerebellum, while decreased glucose metabolism in the lingual gyrus (20), and iRBD with significant decreased occipital cortex glucose metabolism is often associated with mild cognitive impairment (19). Hypermetabolism in the pontine region of the brain in patients with iRBD may be an early alteration in the dystonia regulatory area, and hypermetabolism in the cingulate and hippocampal regions may be due to abnormal neuronal generation and an early alteration in the conversion of iRBD to α-synucleinopathy (21–23). A previous longitudinal study found that all patients with iRBD developed cognitive impairment after 6.4 years, and there was a significant correlation with decreased glucose metabolism in the PVC region, suggesting that decreased metabolism in the PVC region may be responsible for their cognitive impairment (24).

The iRBD metabolic pattern (RBD-related pattern, RBDRP) was determined using ^18^F-FDG-PET and was characterized by increased metabolic activity in the pons, cerebellum, thalamus, hippocampus, medial frontal area, superior limbic gyrus, and inferior temporal gyrus, while decreased metabolic activity in the occipital and superior temporal gyrus. This pattern was significantly expressed in the early stages of PD and correlated with the severity of PD (25). Expression of both PDRP and iRBDRP was higher in patients with a more severe form of PD (PD-MCI), which indicates that expression of the 2 patterns increases with disease severity (26). Therefore, it can predict the future regression of patients with iRBD and has clinical applications.

### Changes in intracerebral blood perfusion

Single photon emission computed tomography is a neuroimaging tool to measure regional cerebral blood flow (rCBF) (27). Using SPECT, it was demonstrated that patients with iRBD had decreased perfusion in the bilaterally frontal, temporal, and parietal lobes, while increased perfusion in the hippocampus, putamen, and pons (28), and the extent of this cerebral perfusion change was more pronounced in patients with iRBD with cognitive impairment (22). However, no correlation was found between changes in local cerebral blood flow and the duration of RBD symptoms in patients (28). Another study found that the cerebral perfusion in the parietal occipital and parietal temporal regions was decreased in patients with iRBD, and this decreased level of perfusion could be used to predict the conversion of iRBD to α-synucleinopathy (29). A longitudinal study found that increased frontal and occipital temporal perfusion returned to normal control levels as the duration of iRBD patients progressed (30), suggesting that compensation may have occurred. We hypothesized that this change in brain perfusion pattern is an intermediate link between iRBD and neurological symptoms and that longitudinal exploration of local cerebral blood flow levels in different brain regions with iRBD can help to understand the pathophysiological mechanisms underlying the transformation to α-synucleinopathy.

## Changes in striatal dopamine transporter protein

The main pathological alteration in PD is degeneration of the substantial nigra-striatal dopaminergic pathway (31). Striatal dopamine levels can be reflected by selective binding of dopamine transporter (DAT) using specific tracers. Several studies have shown that patients with iRBD have abnormal DAT imaging (8, 32–34), and are at high risk for short-term conversion to α-synucleinopathy (35, 36). However, a meta-analysis noted that while abnormal DAT imaging supports the conversion to α-synucleinopathy, there was high heterogeneity among neuroimaging methods and multicenter studies were needed to determine the diagnostic validity of DAT-SPECT (37). The results of a multicenter study that included 1,280 patients with iRBD suggested a 1.98-fold risk of conversion to α-synuclein in patients with iRBD with abnormal DAT imaging at baseline (38), with a low effect size. We speculated that the value of DAT imaging as a predictive marker may have been severely underestimated because only some patients in the study underwent DAT imaging and the imaging methods were not uniform. Notably, multiple predictors identified by the study were non-specific (36), for which the results of the quantitative DAT-SPECT study suggest that reduction of FP-CIT uptake in putamen greater than 25% can predict synucleinopathy during an average follow-up of 3 years (35). A growing body of evidence had demonstrated that abnormal DAT binding can predict the future short-term risk of clinically-defined α-synucleinopathy diagnosis (34). Previous studies showed that iRBD patients with mild cognitive impairment (MCI) who had abnormal DAT manifestations had a higher transformation to α-synucleinopathy risk was higher in iRBD patients with abnormal DAT imaging (HR = 25.05) (39). Therefore, the presence of abnormal DAT imaging in patients with iRBD suggests that patients have developed pathological changes of neurodegenerative disease and their neuronal loss has not yet reached the clinical diagnostic threshold. Therefore, abnormal DAT imaging may be used as a predictor of conversion to α-synucleinopathy.

## Changes in the 5-hydroxytryptaminergic and noradrenergic systems in the brain

Previous studies have confirmed the involvement of the mesencephalic 5-hydroxytryptaminergic and norepinephrinergic systems of the locus coeruleus in the pathophysiology of α-synucleinopathy (40). Thalamic monoaminergic dysfunction was found in patients with iRBD (41), reflecting abnormalities in the terminals of neurons originating from the raphe nucleus and locus coeruleus projections. Although 5-hydroxytryptaminergic reuptake inhibitors can induce RBD symptoms (42), no imaging evidence of impaired an 5-hydroxytryptaminergic system in patients with iRBD has been found (37, 42–44). It was found that the norepinephrine-rich blue spot nuclei in the brainstem of PD patients were abnormally visualized and preceded by dopaminergic (45), which confirms the Braak staging theory of PD, a bottom-up pathological damage process (46). *In vivo* study of noradrenergic changes in 17 patients with iRBD using ^11^C-MeNER as a tracer revealed that tracer uptake levels were decreased in the locus coeruleus to the thalamus and red nucleus regions in patients with iRBD compared to normal controls (47). Compared with PD patients, the uptake level of ^11^C-MeNER tracer in the hypothalamus, red nucleus, and locus coeruleus of PD patients with RBD decreased. Moreover, the abnormal noradrenergic level of PD patients with RBD was positively correlated with abnormal muscle activity during REM sleep (48). Therefore, it was suggested that norepinephrine plays an important role in the pathogenesis of iRBD. A recent study found that the uptake level of ^11^C-MeNER in the primary sensorimotor cortex of iRBD patients decreased (49), and it was speculated that there was nerve fiber projection damage from the locus coeruleus to the primary sensorimotor cortex. These evidences indicated that the norepinephrine system in locus coeruleus may become the target of early intervention of iRBD, but there was still a lack of longitudinal research to prove the application value of norepinephrine.

## Changes in microglia

Microglia are widely distributed immune cells in the central nervous system and are closely associated with neuronal inflammatory responses, and long-term activation of microglia may be involved in the development and progression of neurodegenerative diseases (50). Previous studies had demonstrated that peripheral immunity contributes to PD development (51). Several studies have shown microglial activation in patients with α-synucleinopathy, suggesting that neuroinflammation may be a causal mechanism for α-synucleinopathy (52). Controlling microglia activation in the early stages of the disease could provide a potential therapeutic strategy to slow down disease progression. Using ^11^C-PK11195 as a tracer, an *in vivo* study of 20 patients with iRBD revealed that microglia activation was elevated in the nigrostriatal region and higher than in the striatal region in patients with iRBD compared to normal controls (53), and that elevated levels of ^11^C-PK11195 uptake in the nigrostriatal region correlated with decreased levels of ^18^F-DOPA uptake in the striatum, and that caudate nucleus involvement was consistent with impairment of Lewy body dementia (54), suggesting that synaptic dysfunction of striatal dopamine neurons in patients with iRBD is accompanied by an inflammatory response in the substantia nigra. The observation of microglia activation in the striatal region revealed a significantly higher level of activation than in the substantia nigra (52), probably due to the size of the tissue structure in both regions. Recent studies have found significantly higher levels of microglia activation in the occipital lobe of patients with iRBD (41), suggesting that neuroinflammation in the occipital lobe is involved in the pathogenesis of iRBD. However, whether the level of microglia activation in iRBD can be used as a biomarker for conversion to α-synucleinopathy needs to be further investigated and demonstrated due to the lack of relevant studies.

## Changes in cardiac sympathetic never function

An increasing number of clinical studies have focused on the evaluation of cardiac sympathetic nerve function in iRBD. [^123^I] metaiodobenzylguanidine (^123^I-MIBG) cardiac imaging is a commonly used imaging technique to diagnose cardiac sympathetic function (55). It was found that ^123^I-MIBG uptake levels were decreased in the heart of PD patients (56), and more significantly in PD patients with RBD (57). Studies have shown that patients with iRBD also have decreased ^123^I-MIBG uptake levels (58, 59), and are similar to Lewy body dementia. Previous studies had found that cardiac sympathetic function was linked with the severity of REM in iRBD patients (60). Furthermore, some studies have shown that abnormal ^123^I-MIBG manifestations can precede the dopaminergic system in patients with iRBD (61, 62). However, a longitudinal study of iRBD patients followed for 2.8 years found no significant changes in the level of ^123^I-MIBG uptake by the patient's heart, possibly related to the course of the disease (57), which was contrary to the findings of other studies (63). Despite the high sensitivity and specificity of early autonomic damage in PD to cardiac sympathetic imaging, whether ^123^I-MIBG can be a neurobiological marker to predict disease progression in patients with iRBD needs to be further validated (64).

## Conclusion

Neuroimaging reveals the presence of structural and functional alterations in iRBD that precede α-synucleinopathy (Table 1). The use of molecular imaging techniques may contribute to what may be a sensitive screening tool for exploring iRBD-transformed α-synucleinopathy, with a wide range of clinical applications. Compared to other risk factors for α-synucleinopathy, such as cognitive deficits, olfactory impairment and dopamine transporter protein (DAT-SPECT) abnormalities are at much higher predicted risk in the conversion of iRBD to α-synucleinopathy. Therefore, a single imaging modality for iRBD does not yet fully reflect the underlying pathophysiological changes in iRBD or the different clinical features. Therefore, adopting multiple complementary neuroimaging examinations and conducting prospective studies to validate the search for sensitive and effective biomarkers of iRBD conversion to α-synucleinopathy will help to accurately identify the clinical conversion of iRBD.

**Table 1:** Main findings of each neuromolecular imaging studies in iRBD patients.

**Authors [Ref]**	**Method**	**RBD/controls**	**Results and conclusion**
Iranzo et al. (65)	^123^I-FP-CIT-SPECT	20 iRBD/ 20 control participants	• ^123^I-FP-CIT-SPECT can monitor the progression of the nigrostriatal damage in iRBD patients. • Nigrostriatal dopaminergic dysfunction can predict the conversation from iRBD to PD after 3 years.
Iranzo et al. (35)	^123^I-FP-CIT-SPECT	87 iRBD/20 control participants	• Reduction more than 25% in the putamen can discriminate iRBD patients who converted to a synucleinopathy after 3 years.
Iranzo et al. (66)	^123^I-FP-CIT-SPECT	43 iRBD/18 control participants	• Hyperechogenicity of the substantia nigra and lower striatal presynaptic DAT can accurately predict the conversion of iRBD patients to a synucleinopathy after 2.5 years.
Li et al. (36)	^99m^TC-TRODAT-1 SPECT	43 iRBD	• Decreased DAT in the putamen and striatum had the predictive value after 5 years.
Dang-Vu et al. (21)	^99m^Tc-ECD SPECT	20 iRBD	• iRBD patients who converted to a synucleinopathy had increased perfusion in the hippocampus.
Holtbernd et al. (67)	^18^F-FDG PET	10 iRBD/10 control participants	• Latent network abnormalities in iRBD patients were associated with the probability of conversion.
Kogan et al. (68)	^18^F-FDG PET	20 iRBD	• PD-related brain pattern will be used to as a prodromal PD biomarker after 3.7 years.
Arnaldi et al. (69)	^123^I-beta-CIT-SPECT	263 iRBD/243 control participants	• Constipation, age over 70 years, and putamen dopaminergic dysfunction will the best combination of risk factors to predict conversion.
Janzen et al. (70)	^123^I-MIBG	37 iRBD	• 78.4% iRBD patients showed a pathological ^123^I-MIBG. • Combination hyposmia and pathological ^123^I-MIBG can identify iRBD patients in an early prodromal stage of PD/DLB after 4 years.

## Author contributions

CG: wrote first draft. HZ: conceptualization and resources. All authors approved the submitted version.

## Funding

This work was supported by the Henan Medical Science and Technology Research Program (No. 202102310082), and Henan Province Medical Science and Technology Tackling Provincial Ministry Key Projects (SBGJ202102033).

## Conflict of interest

The authors declare that the research was conducted in the absence of any commercial or financial relationships that could be construed as a potential conflict of interest.

## Publisher's note

All claims expressed in this article are solely those of the authors and do not necessarily represent those of their affiliated organizations, or those of the publisher, the editors and the reviewers. Any product that may be evaluated in this article, or claim that may be made by its manufacturer, is not guaranteed or endorsed by the publisher.
